# Supplementary material for: The Prevalence and Molecular Landscape of Lynch Syndrome in the Affected and General Population
Source: Cancers (Basel). 2023 Jul 18;15(14):3663. doi: 10.3390/cancers15143663 (PMC10377710; doi:10.3390/cancers15143663)
Supplement: Supplementary file 1 [file cancers-15-03663-s001.zip › cancers-2476781-supplementary.pdf]

Supplementary data

Table S1. Details of EstBB cohorts.

	Total	Not enough detailed data	Healthy or benign changes	One cancer in health history	Two cancers in health history	Three or more cancers in health history	Most frequent cancer site	Mean age of first cancer	Mean age of LS diagnosis	MMR IHC done
**EstBB cohort, not yet recalled (n=37), deceased (n=2)**	39	0	32	2	1	4	Skin	52.1 years	NA	NA

**EstBB cohort, recalled (n=30), deceased (n=2)**	32	0	24	3	2	3	Skin	52.1 years	NA	NA
**EstBB cohort *MLH1* carriers, recalled (n=8), deceased (n=1)**	9	0	4	1	1	3	Colon	43.0 years	NA	NA

Table S2: MMR gene variants represented in EstBB cohorts

Gene	Variant	No of individuals carrying this variant	Exon/Intron position	Class of variant based on ACMG Criteria
**NM_000249.4(MLH1)**				
MLH1	c.1976G>C, p.(Arg659Pro)	9	17	Pathogenic
MLH1	c.1668-1G>T, p.?	2	intron 14	Likely pathogenic
**NM_000251.3(MSH2)**				
MSH2	c.793-1G>A, p.?	8	intron 4	Pathogenic/likely pathogenic
MSH2	c.181C>T, p.(Gln61*)	1	1	Pathogenic
MSH2	c.2131C>T, p.(Arg711*)	2	13	Pathogenic
**NM_000535.7(PMS2)**				
PMS2	c.943C>T, p.(Arg315*)	16	9	Pathogenic
PMS2	c.825A>G, p.(Gln275=)	13	8	Pathogenic/likely pathogenic
PMS2	c.861_864del, p.(Arg287fs)	14	8	Pathogenic
**NM_000179.3(MSH6)**				

MSH6	c.1095G>A, p.(Trp365*)	1	4	Pathogenic
MSH6	c.3226C>T, p.(Arg1076Cys)	14	5	Likely pathogenic
